# Broncho-pleuro-cutaneous fistula and pneumothorax: Rare challenging complications of chest wall eumycetoma

**DOI:** 10.1371/journal.pntd.0005737

**Published:** 2017-09-28

**Authors:** Eiman Siddig Ahmed Saad, Ahmed Hassan Fahal

**Affiliations:** The Mycetoma Research Centre, University of Khartoum, Khartoum, Sudan; Saudi Ministry of Health, SAUDI ARABIA

## Case presentation

We report on a 60-year-old housewife who presented to the Mycetoma Research Centre (MRC) at the University of Khartoum on the 7 December 2016 with a 30-year history of chest wall eumycetoma due to *Madurella mycetomatis*. Two months prior to presentation to the MRC, a discharging sinus in the left lateral side of the chest wall was noted by the patient. The discharge was purulent and contained black grains. Just before presentation, air leak from the sinus was noted and she developed general weakness and deterioration.

The patient was noncompliant with ketoconazole therapy for eumycetoma and has had 3 previous surgical excisions of her chest wall lesion; the first one was in 1991, the second one was in 1995, and the third excision was done in 2002. However, she ceased treatment and follow-up in 2002.

The patient has had diabetes mellitus for 14 years and hypertension for 1 year. She has various diabetic microvascular complications, diabetic nephropathy, and diabetic septic foot, leading to left above-knee amputation in 2015, and she later developed right gangrenous middle and fourth toes. Her drug history includes insulin 10/5 units, furosemide 40 mg, aspirin 81 mg, atorvastatin 20 mg, and amlodipine 10 mg per day. She has had multiple hospital admissions and blood transfusions due to chronic kidney disease secondary to diabetic nephropathy.

She is a housewife with a low socioeconomic status. A family history of mycetoma was noted; her son has bilateral lower limb eumycetoma.

**Fig 1:**
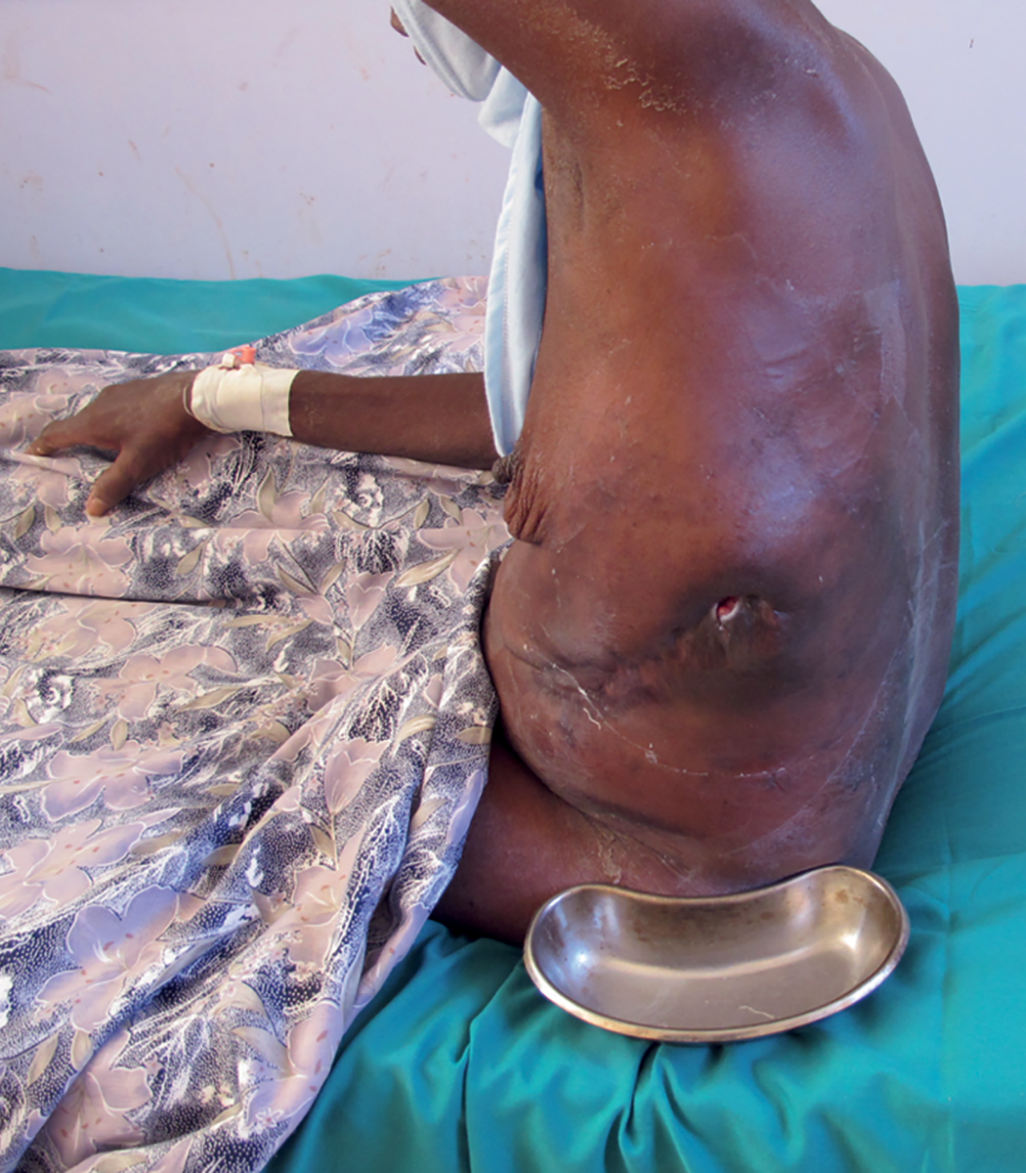
Photograph of the patient showing the chest wall lesion with the sinus.

Clinically she looked unwell and pale. She was haemodynamically stable, with a pulse rate of 72 beats per minute, respiratory rate of 20 breaths per minute, and blood pressure of 130/70 mmHg. Her head and neck examinations were unremarkable. A respiratory examination showed no signs of respiratory distress, and the trachea was central, but the movement of the left side of the chest was reduced. A 2 cm in diameter sinus discharging an exudative material with black grains was noted in the midaxillary line of the left chest. The discharge was noted to increase with cough (Fig 1). There was a decrease in air entry and stony dullness on the left side but no added sounds. Other systems were unremarkable.

Investigations showed a haemoglobin level of 5.0 g/dl with normal leucocyte count and platelets. Her random blood glucose level was elevated at 324 mg/dl. There was evidence of chronic kidney disease, with urea 94 mg/dl and creatinine 3.2 mg/dl. She was hyponatremic with a serum sodium of 129 mmol/l and a normal K^+^ of 4.6 mmol/l. A liver function test showed an elevated alkaline phosphatase (ALP) of 228 IU/l; otherwise, other enzymes were normal, with aspartate aminotransferase (AST) 6 IU/l and alanine aminotransferase (ALT) 4 IU/l. Total protein was normal at 6.9 g/dl, but a low serum albumin (1.8 g/dl) was noted. C-reactive protein was 11 mg/l, and a viral screening was negative. A urinalysis was positive for pus cells of 8–12/hpf, albumin 1+, and sugar 2+. After the stimulation of whole blood with culture filtrate of *M*. *mycetomatis*, cytokines interleukin (IL)-10 and interferon gamma (IFN-γ) levels were measured. The levels of these cytokines were higher in the patient compared with control subjects. This indicates a mixed type of immune response in which both Th1 cytokines and Th2 cytokines are secreted at the same time.

**Fig 2:**
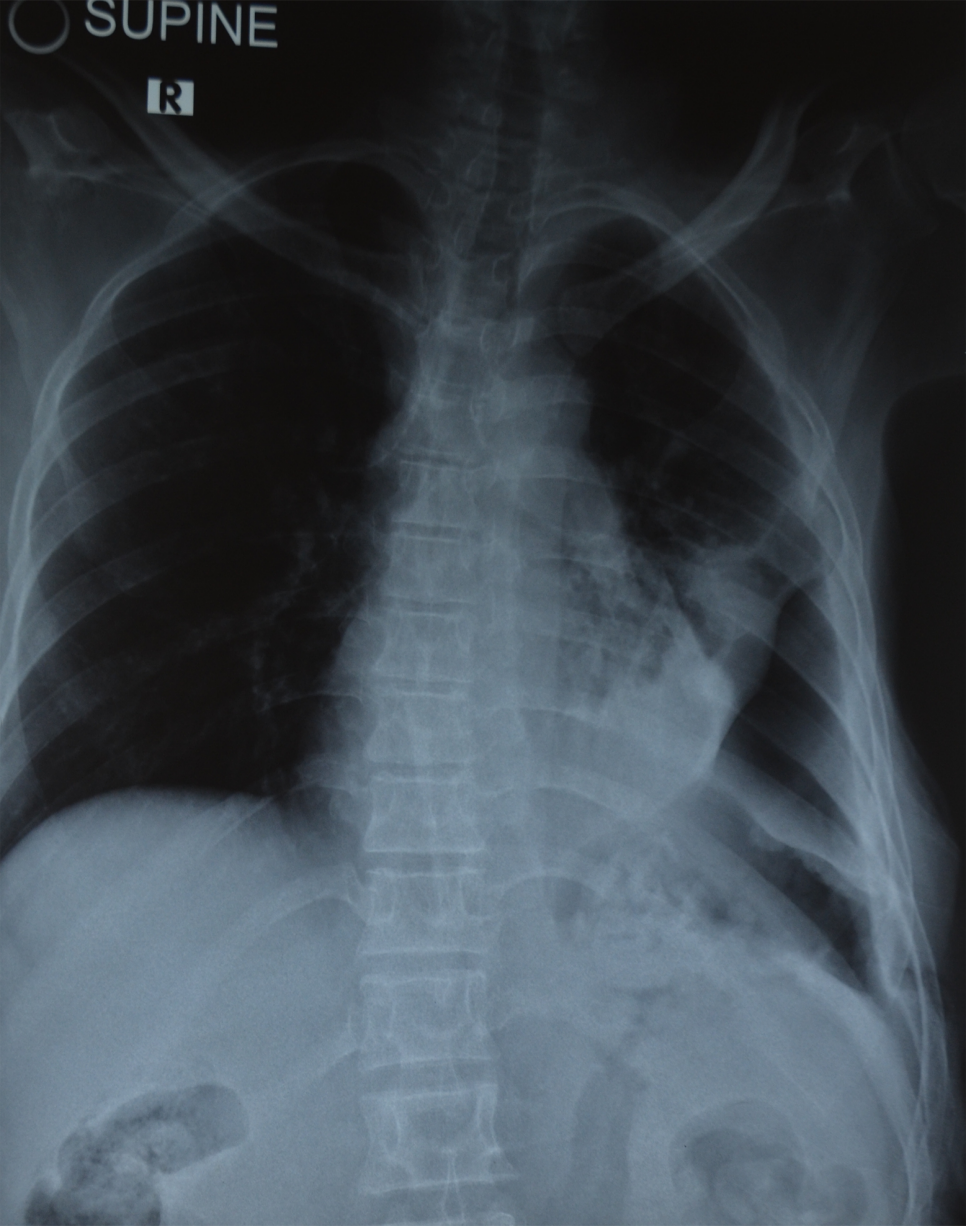
Chest X-ray showing soft tissue in left lateral part of the chest wall, left lower lung mass collapse, decrease lung mass with left-sided pneumothorax.

She underwent imaging investigations, including chest X-ray, computed tomography (CT), and MRI scan examinations. The chest X-ray revealed soft tissue in the left lateral part of the chest wall, a left lower lung mass, reduced lung volume, and left- sided pneumothorax (Fig 2).

**Fig 3:**
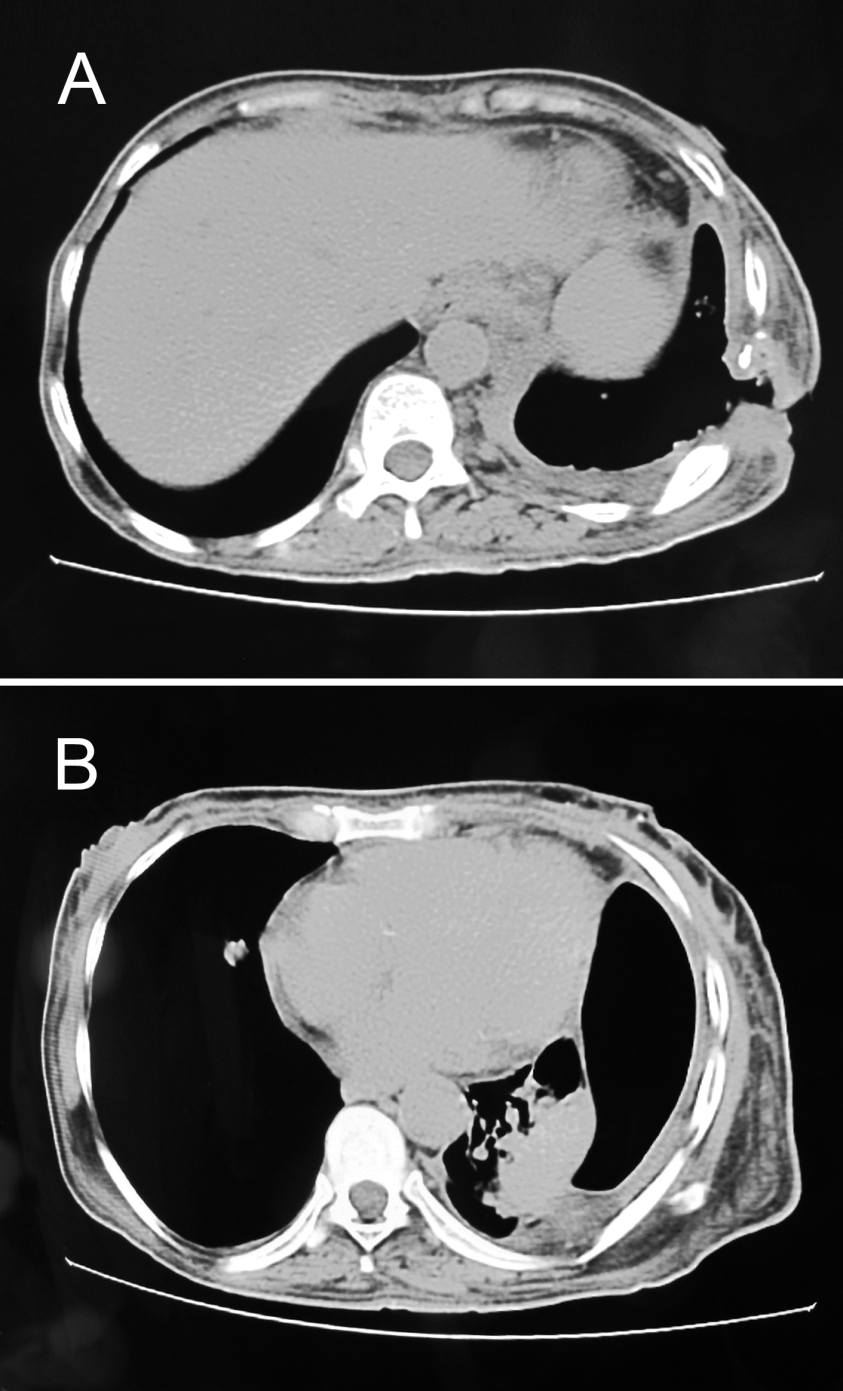
Chest computed tomography (CT) scan showing (A) left lower chest wall thickening with a wall defect and (B) left side hydropneumothorax with lower lobe collapse and consolidation of the lingual.

A CT scan showed a left lower chest wall thickening with a wall defect, left side hydropneumothorax, and lower lobe collapse and consolidation of the lingual (Fig 3).

**Fig 4:**
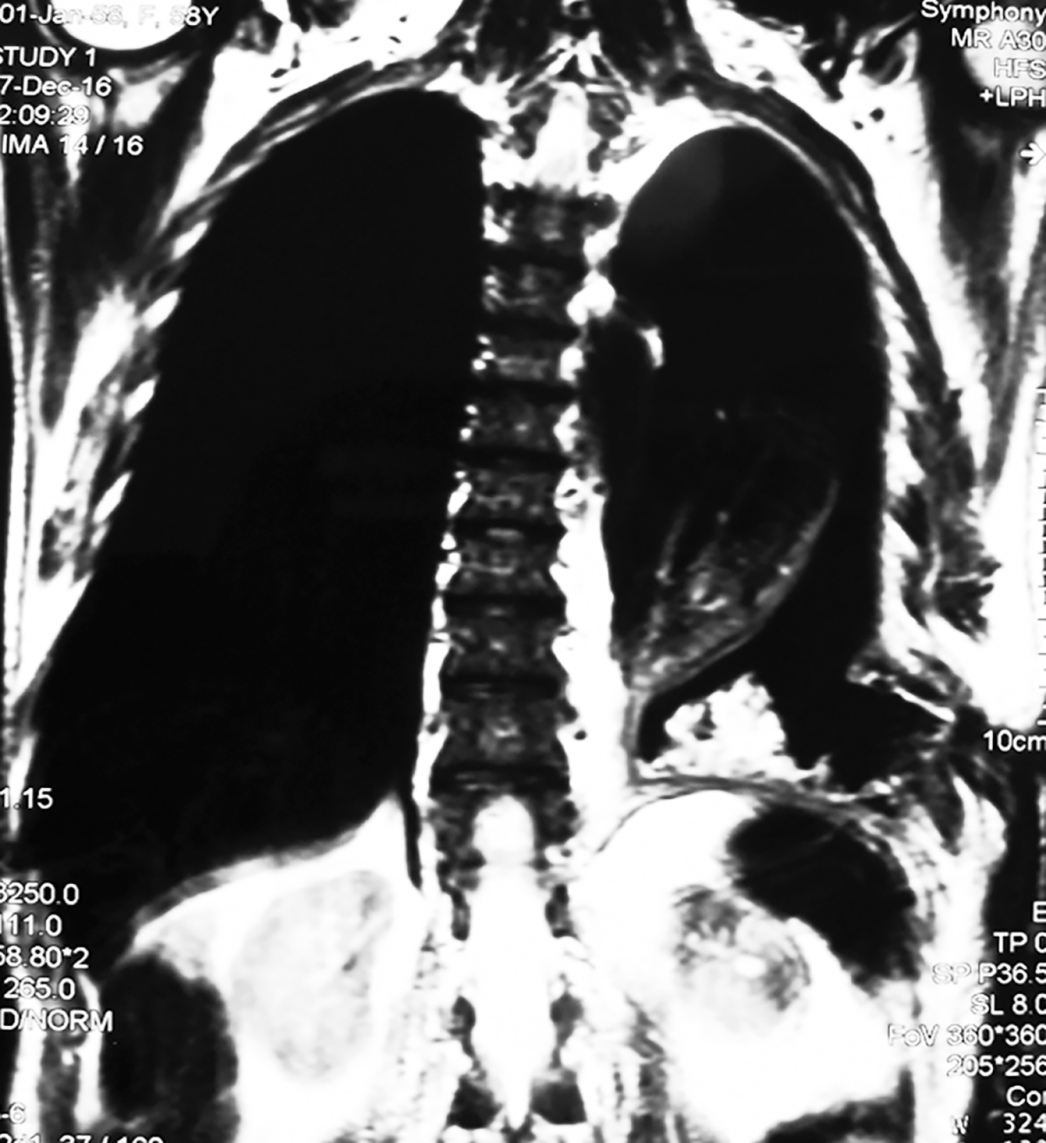
Chest MRI showing marked volume loss in left hemithorax, pneumothorax, soft tissue lesion in the left lingular region, left lower lobe atelectasis, and a small amount of pleural effusion.

An MRI examination revealed marked volume loss in the left hemithorax, pneumothorax, soft tissue lesion in the left lingular region, left lower lobe atelectasis, and a small amount of pleural effusion (Fig 4).

A grains culture was done on Sabouraud agar, and it revealed evidence of *M*. *mycetomatis*. A molecular diagnosis of causative organism was done by a grains PCR examination, and it confirmed the diagnosis of *M*. *mycetomatis*.

She was admitted to the hospital and received 5 units of blood to correct the anaemia, as well as 18 units of albumin. A swab culture from the sinus discharge revealed *Escherichia coli*, which is sensitive to imipenem and amikacin. Imipenem 500 mg twice a day was prescribed for 5 days. An intercostal chest drain was inserted to drain the intrathoracic fluid collection. Treatment for eumycetoma was commenced with itraconazole 400 mg per day. Her condition showed a gradual improvement, with a plan to stay on antifungal treatment. Given her poor general condition, malnutrition, and chronic kidney disease, surgical exploration was deferred. She was seen in April of 2017 at the clinic. Her condition is stable, and the secondary infection is reduced, but the fistula is still open.

## Case discussion

Mycetoma is one of the most destructive neglected tropical diseases, characterised by the formation of a chronic inflammatory granuloma, caused either by fungal (eumycetoma) or bacterial (actinomycetoma) microorganisms. If left untreated, it will induce massive deformities, destructions, and disabilities. It has enormous damaging effects on patients, family, and community in endemic regions [1,2,3].

In mycetoma, the foot and hand are the most affected sites. The chest wall is a rare site, accounting for 0.1% of 6,792 mycetoma patients seen at the MRC in the period 1991–2014 [4,5]. Only 2 patients with mycetoma-induced pulmonary complications were reported [6,7].

The triad of subcutaneous mass, sinuses, and discharge-containing grain is pathognomonic of the disease [8,9]. It is interesting to note that our reported patient presented with a single large sinus but a small subcutaneous mass. However, most of the mass was intrathoracic, which is an uncommon finding. The late presentation of this patient is multifactorial, including the painless nature of the disease, poor health education, low socioeconomic status, and dissatisfaction with the available antifungal treatment, and hence health education among the affected communities is highly recommended.

A broncho-pleuro-cutaneous fistula is potentially fatal; however, in the reported patient, the process was slowly progressive, with the formation of a large intrathoracic granuloma with thick capsule leading to lung volume reduction and encapsulated pneumothorax. The formation of fistula in this patient is likely to be due to the long-standing progressive disease, diabetes, and its complications and due to her age. Surgical repair of the fistula is the treatment of choice in such patients. However, the large intrathoracic granuloma, her poor general condition, and her chronic kidney disease hindered the surgical intervention [10].

A search of the medical literature failed to reveal any report on such presentation, and hence it is worth reporting this patient presentation in order to share the experience.

## Ethics statement

Written informed consent was obtained from the patient.

**Key learning points**

- Chest wall eumycetoma is an unusual condition.
- The reported intrathoracic pathology is rare.
- Chest CT scan and MRI are essential to confirm the diagnosis.
- Management of such presentation is challenging.
- Health education and early presentation are crucial to avoid such presentation.
